# Three dimensional (3D) gingival models in periodontal research: a systematic review

**DOI:** 10.1007/s10856-023-06761-z

**Published:** 2023-11-08

**Authors:** Z. AlFatlawi, M. Huang, D.Y.S. Chau, F. D’Aiuto

**Affiliations:** 1 Periodontology Unit, UCL Eastman Dental Institute, 21 University Street, London, WC1E 6DE UK; 2 Division of Biomaterials and Tissue Engineering, UCL Eastman Dental Institute, Royal Free Campus, Rowland Hill Street London, NW3 2PF UK

## Abstract

The aim of this study is to systematically appraise the evidence on available full thickness 3D gingival and mucosal models (3D culture in scaffold base system) and their application in periodontal and peri-implant research. This study involved a systematic review of twenty-two studies obtained from searching from five electronic databases: MEDLINE-OVID, EMBASE, EBSCOhost, Web of Science Core Collection and LILACS, as well as a hand search of eligible articles up to September 2022. A total of 2338 studies were initially identified, after removal of duplicates (573), abstracts/title selection (1765), and full text screening (95), twenty-two studies were included, thirty-seven models were identified. Several cellular markers were reported by the studies included. The expression of keratinocytes differentiation markers (K4, K5, K10, K13, K14, K16, K17, K18, K19, involucrin, laminin5), proliferation marker (Ki67, CD90), and vimentin, Type I, II and IV collagen produced by fibroblasts were investigated in thirty models. No quantitative analyses were performed, and results of the review confirmed a substantial level of heterogeneity across experiments. In conclusion, there is currently insufficient evidence to conclude that the available 3D gingival and mucosal models can entirely recapitulate the human gingival tissue/mucosa and provide a useful research tool for periodontal and peri-implant research. This review also highlighted the lack of a standardized protocol to construct and characterize 3D gingival models. A new protocol is proposed for the characterization of in vitro gingival models for future research.

## Introduction

For several years, two-dimensional (2D) cell cultures as an in-vitro tool, and animal models have been commonly used in periodontal research to study: disease patho-mechanisms, test new therapeutics and evaluate new regenerative strategies [1, 2]. 2D cell culture and animal models, however, are not free from limitations. For example, a 2D cell culture of gingival cells cannot fully replicate the architecture, physiological, and pathological microenvironment of living human gingival tissue, plus ethical and financial concerns are associated with animal experiments [3].

Three-dimensional (3D) gingival models provide researchers with an alternative to animal experimentation and 2D cell culture. Studies have reported the construction of 3D gingival models since 1997 with modified cell sources, scaffolds, and culture media. Initially, partial thickness models were constructed including epithelial tissue in absence of underlying connective tissue or connective tissue including gingival fibroblast cells without epithelial components [4, 5]. To date, full thickness 3D gingival models using human gingival-derived cellular sources including keratinocytes to assemble the epithelial layer and human gingival fibroblasts to establish the connective tissue layer are available. The advantage of full thickness 3D gingival model is their closer recapitulation to the complex structures and functions of native human gingival tissue [6, 7]. Several studies have demonstrated the application of these models in periodontal research. For instance, Dabija-Wolter et al. demonstrated the using of 3D gingival model to study host-microbial interaction. In this study, they examined the extent destruction of epithelial layer due to invation of F. nucleatum. They concluded invation of this pathogenic bacteria will trigger elimination of bacterial infection through epithelial shredding without causing a permanent damage of the tissue in 3D gingival model [8]. Razali et al. used 3D peri-implant model to understand the effect of photofunctionalization on three different types of implant abutment materials (yttriastabilized zirconia, alumina-toughened zirconia, and grade 2 commercially pure titanium). They and concluded that photofunctionalization of implant abutment materials improved the biological seal of the surrounding soft tissue peri-implant interface [9].

Although growing evidence have shown the promising outcome of 3D gingival model in periodontal research, there’s no consensus on fabrication method and material neither ideal characteristics for 3D gingival model. Studies have suggested that to recapitulate native gingival tissue, 3D model should be consisted of epithelial and connective tissue layers, which were separated by well define basement membrane. In addition, differentiation markers of each cell component, and functional assessment of the layers are also crucial [10, 11]. However, a critical evaluation of all these different types of models is missing. Indeed, all these different types of gingival models have not been reviewed with regards to their representation of human gingival tissue. The aim of this study was therefore to appraise current available 3D in vitro gingival models constructed using organoid cell culture system and provide answers to the following questions:

1. Are any of the current 3D gingival models better replicate the native human gingival tissue in terms of their structure, differentiation characteristics, and barrier function.
2. What are the available substrates that are used to reconstruct 3D gingival models?

## Materials and methods

### Focused questions

In view of the lack of specific tools to define the specific research questions we adapted the PICOS tool to search systematically for available evidence.

(P)Participant: 3D cell culture gingival model that is constructed by seeding gingival fibroblasts cells in the substrate and co-cultured with oral epithelial cells.

(I/E) Type of intervention/Exposure: N/A.

(C) Comparison: native human gingival tissue.

(O) Outcomes:

- 1-Resemblance of native human gingival tissue (3D structural layers evaluated by
- histological analysis)
- 2- Differentiation markers of each cell component.
- 3- Functional assessment of the layers

(S) Studies type: In vitro experiments.

### Protocol registration and reporting format

A systematic review protocol was developed and registered with the Open Science Framework (OSF) database, hosted by the Center for Open Science(COS) (https://archive.org/details/osf-registrations-6mzw2-v1 - License: http://www.gnu.org/licenses/lgpl-3.0.txt). Further when possible the systematic review was conducting according to the PRISMA guidelines [12].

### Search strategy

Five electronic databases: MEDLINE (OVID), EMBASE, Dentistry and Oral Science Source (EBSCOhost), Web of Science Core Collection and LILACS (Latin American & Carribbean Health Sciences Literature) were included and updated up to the 12th of September 2022.

Hand searching process was performed by 2 independent reviewers (ZA and MH) and in case of any dispute further discussion with a third reviewer occurred (FDA). Only studies in the English language were included.

### Study selection

All articles retrieved were exported and de-duplicated using the Reference Management Software “EndNote X9.3.3 (Bld 13966)”.

#### Study eligibility assessment

Screening and assessment of study eligibility were performed by 2 reviewers independently (ZM & MH) according to the inclusion and exclusion criteria. Agreement between the 2 reviewers was determined by kappa statistics.

##### Inclusion/exclusion criteria

*Inclusion Criteria:*

- Studies of 3D cell culture gingival models constructed with a substrate seeded by human gingival fibroblasts or human periodontal ligament cells and human gingival/oral epithelial cells
- 3D cell culture gingival model construct with scaffold base system
- Including histological analysis
- Published in the English language.

*Exclusion Criteria:*

- 3D cell culture gingival model which was constructed without substrate base system
- Studies of 3D cell culture gingival model which constructed with a substrate that seeded by non-human sources of fibroblast or epithelial cells.
- Studies of 3D cell culture gingival model which was constructed with a substrate that seeded by human gingival fibroblasts or human periodontal ligament cells without human gingival/oral epithelial cells.
- Studies of 3D cell culture gingival model which was constructed with a substrate that seeded by human gingival/oral epithelial cells without human gingival fibroblasts or human periodontal ligament cells.
- Animal studies.
- Studies without clear histological analysis.
- Abstracts without full papers.

#### Data extraction strategy

Piloting of data extraction was conducted before starting with the full search strategy, further as some articles had a different methodology to prepare 3D models other than human cell sources two reviewers (ZM & MH) performed pilot runs using a specially designed data extraction spreadsheet. Any disagreements were resolved by discussion and if this was not possible, arbitration with an experienced reviewer was considered (FDA). Main categories of data were extracted as listed below: Study Characteristics Data: “Study authors, Year of publication and title, Study design, Conclusions”, “Participant/ 3D cell culture gingival model with inclusion/exclusion criteria, Human gingival fibroblasts cells, Specific substrate for cells seeding, Human epithelial cells “.

### Study bias protection assessment

Quality assessment of included trials undertaken independently and in duplicate by two reviewers (ZM & MH) as part of the data extraction process. There are no established criteria for evaluating in vitro studies. Two tools of risk of bias were used in this review. The first one was the modified ARRIVE guidelines (Supplemental Data 2) to assess the quality of each study [13]. A second tool ‘Systematic Review Centre for Laboratory Animal Experimentation (SYRCLE)’s risk of bias tool’ was also used to analyze data and adapted by ruling out the blind intervention section [14].

## Results

### Study selection

**Fig. 1:**
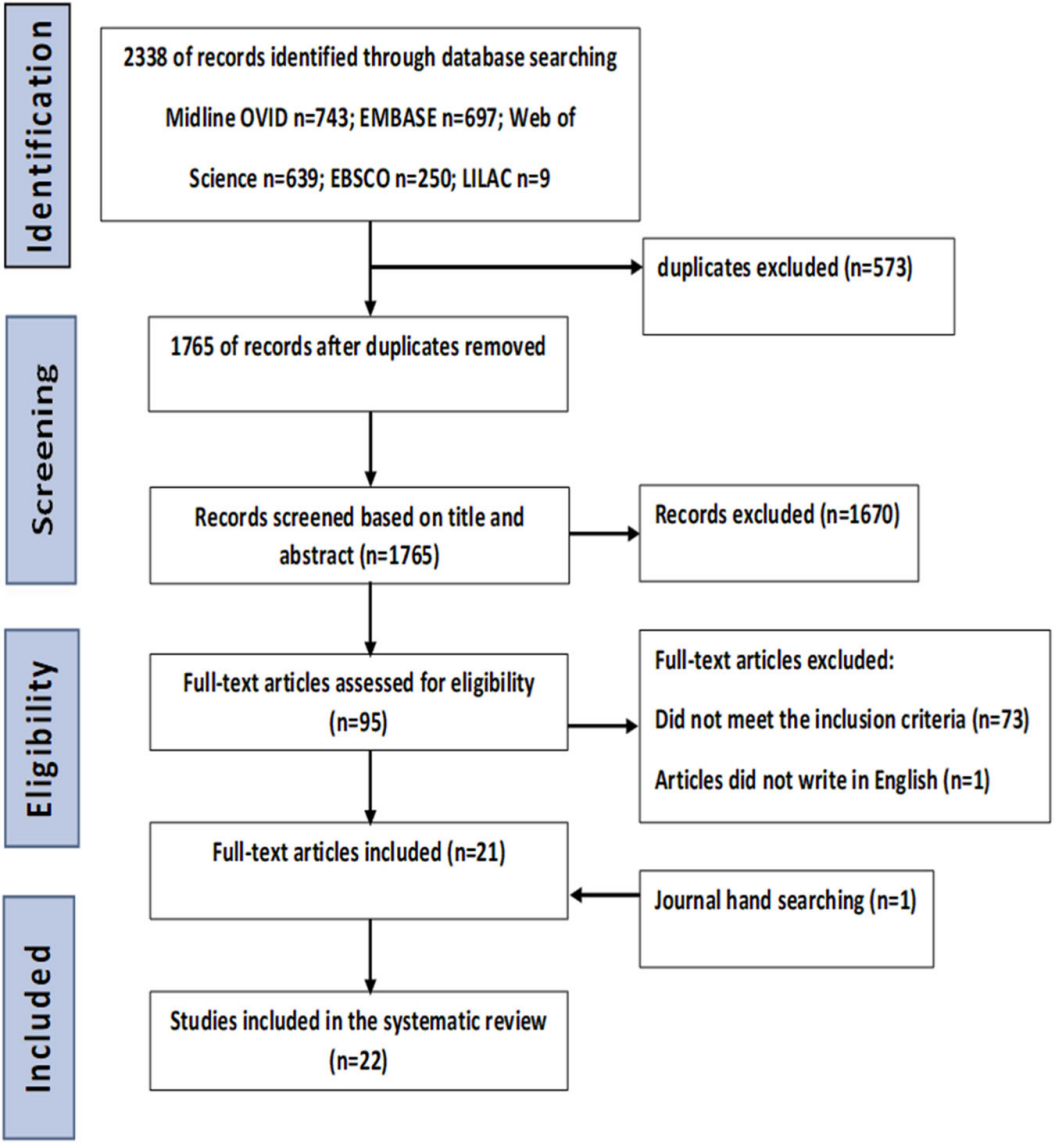
PRISMA flow diagram of the study inclusion process

A total of 2338 articles were identified through database searching and Midline OVID *n* = 743; EMBASE *n* = 697; Web of Science *n* = 639; EBSCO *n* = 250; LILAC *n* = 9. The final number retrieved after completing the selection process was 22 (Fig. 1). Due to the absence of relevant quantitative measures to evaluate gingival models, quantitative models, and meta-analysis were not possible. Qualitative analyses of the evidence retrieved was conducted to summarize the characteristics of 3D gingival models.

### Quality of studies

#### Modified ARRIVE guidelines (Supplemental Data 2)

Most of the selected studies were of high quality based on modified ARRIVE guidelines. Only seven studies discussed the scientific implications and limitations [9, 15–20].

Five studies did not give the statement of potential conflicts and funding disclosure [21–25] while one article was not published in a peer reviewed journal [26].

#### SYRCLE bias assessment

Well-balanced results in terms of low, unclear, and high risk of selection bias across studies were identified. All studies presented with high risk of bias in the random sequence generation and baseline variable characteristics. On analyzing allocation concealment, most selected articles had an unclear risk of bias, and only two articles had a low risk of bias [25, 27]. The randomization parameter was at high risk of bias. On analyzing random outcome assessment, all studies had an unclear risk of bias. In addition, all articles presented a low risk of bias in the results of incomplete outcome data, selective outcome reporting and other sources of bias (Tables 1 and 2).

**Table 1:** Quality assessment and risk of bias (modified from the ARRIVE and CONSORT guidelines)

Studies	1	2	3	4	5	6	7	8	9	10	11	12
[10]	1	1	3	2	3	2	2	3	3	1	1	1
[24]	1	1	3	2	3	2	2	3	3	1	0	1
[19]	1	2	3	2	3	2	2	3	3	1	1	1
[9]	1	2	3	2	3	2	2	3	3	2	1	1
[25]	1	1	3	2	3	2	2	3	3	1	0	1
[15]	1	2	3	2	3	2	2	3	3	1	1	1
[34]	1	2	3	2	3	2	2	3	3	1	1	1
[35]	1	1	2	2	3	2	2	3	3	1	1	1
[21]	1	2	3	2	3	2	2	3	3	1	1	1
[33]	1	2	3	2	3	2	2	3	3	1	0	1
[26]	1	1	3	2	3	2	2	3	3	1	1	0
[8]	1	1	3	2	3	3	2	3	3	1	1	1
[22]	1	2	3	1	3	2	2	3	3	1	0	1
[27]	1	2	3	2	3	2	2	3	3	1	1	1
[17]	1	2	3	2	3	3	2	3	3	1	1	1
[20]	1	1	3	2	3	3	2	3	3	1	1	1
[29]	1	2	3	2	3	3	2	3	2	1	1	1
[23]	1	1	3	2	3	3	2	3	2	1	0	1
[28]	1	2	3	2	3	3	2	3	3	1	1	1
[18]	1	2	3	2	3	3	2	3	3	2	1	1
[30]	1	2	3	2	3	3	2	3	3	1	1	1
[16]	1	2	3	2	3	3	2	3	3	1	1	1

**Table 2:** Quality assessment and risk of bias (SYRCLE tool), each item was scored as “yes”, “no”, or “unclear”

Studies	Selection bias			Performance bias		Detection bias		Attrition bias	Reporting bias	Other bias
	Random sequence generation	Baseline characteristics	Allocation concealment	Random housing	Blinding	Random assessment outcome	Blinding	Incomplete outcome data	Selective outcome reporting	Other sources of bias
[10]	No	Yes	Unclear	Yes	Unclear	No	No	No	Yes	Yes
[24]	No	Yes	Unclear	Yes	Unclear	No	No	No	Yes	Yes
[19]	No	Yes	Unclear	Yes	Unclear	No	Unclear	No	Yes	Yes
[25]	No	Yes	Yes	Yes	No	No	Unclear	No	Yes	Yes
[9]	No	Yes	Yes	Yes	Yes	No	No	No	Yes	Yes
[15]	No	Yes	Unclear	Yes	Unclear	No	Unclear	No	Yes	Yes
[34]	Yes	Yes	Unclear	Yes	Yes	No	No	No	Yes	Yes
[35]	No	Yes	Unclear	Yes	Unclear	No	No	No	Yes	Yes
[21]	No	Yes	Unclear	Yes	Unclear	No	No	No	Yes	Yes
[33]	No	Yes	Unclear	Yes	Unclear	No	No	No	Yes	Yes
[26]	No	Yes	Unclear	Yes	Unclear	No	No	No	Yes	Yes
[8]	No	Yes	Unclear	Yes	Unclear	No	No	No	Yes	Yes
[22]	No	Yes	Unclear	Yes	No	No	No	No	Yes	Yes
[27]	No	Yes	Yes	Yes	Unclear	No	Unclear	No	Yes	Yes
[17]	No	Yes	Unclear	Yes	Unclear	No	No	No	Yes	Yes
[20]	Yes	Yes	Unclear	Yes	Unclear	Unclear	No	No	Yes	Yes
[29]	Yes	Yes	Unclear	Yes	Unclear	Unclear	No	No	Yes	Yes
[23]	Yes	Yes	Unclear	Yes	Unclear	Unclear	No	No	Yes	Yes
[28]	No	Yes	Unclear	Yes	Unclear	No	No	No	Yes	Yes
[18]	No	Yes	Unclear	Yes	Unclear	No	No	No	Yes	Yes
[30]	No	Yes	Unclear	Yes	Unclear	No	No	No	Yes	Yes
[16]	No	Yes	Unclear	Yes	Unclear	No	No	No	Yes	Yes

Individual risk of bias each item in the SYRCLE tool was scored as “yes”, “no”, or “unclear”

### 3D Gingival model characteristics

Up to thirty-seven gingival and peri-implant models were described in the included twenty-two studies. Thirty-six models were constructed using the organotypic culture technique in a static cell culture condition. Only one study used a dynamic perfusion bioreactor system, where disc shape collagen sponge scaffolds were fitted in a perfusion bioreactor [22].

Regarding the cellular source, different types of cells were used including primary cells from gingival tissue biopsies or immortalized cell lines or a combination of both (Table 3).

**Table 3:** Summary of cellular sources used in construction of gingival or peri-implant models

Cells origin	Type of cells		Type & no. of models		References
	Keratinocyte	Fibroblast	Gingiva	Peri-implant	
Primary cells	Primary	Primary	13	5	[8–10, 18, 23–25, 28–30, 35]
Immortilized cells	OKG4/bmi1/TERT	Fib-TERT, T0026	3	1	[17, 20, 25, 27]
	KC-HPV	Fib-TERT, T0026	1		[25]
	HGEK-16	GFB-16	2		[22, 34]
	Gie-No3B11	hTERT	1		[21]
	hTERT (TIGKs, CRL-3397, ATCC)	hTERT (hGFBs, CRL-4061, ATCC)	4		[16]
Primary and Immortilized cells	OKF6/TERT-2	Primary	2	1	[10, 15]
	TR146	Primary		1	[19]
	NOK-si	Primary	1		[33]
	FNB6-TERT	Primary	1		[26]
	H357	Primary	1		[26]

Among these twenty-two studies, only six studies examined human gingival biopsy as a control [16, 25, 26, 28–30].

#### Macroscopical model appearance

In this review, one study by Koskinen Holm, C., & Qu, C. investigated macroscopical appearance of three gingival models constructed by using collagen type I (rat tail) that crosslinked with genipin, cytochalasin D, and genipin/ cytochalasin D, respectively [16]. Genipin is a chemical crosslinking agent, while cytochalasin D, is used to inhibit the rapid actin polymerization [31, 32]. This study showed that the crosslinked models using genipin or genipin/cytochalasin D were larger size in compared with non-crosslinked model.

### Histological analysis

The included studies performed histological structure analysis to evaluate the successful construction of 3D model by using different types of staining techniques such as hematoxylin (H), hematoxylin and eosin (H&E), (H&E) and Periodic acid-Schiff (PAS), Masson’s trichrome, and van Gieson.

#### Epithelium layer

The number of epithelial cell layers was reported in nine studies with thirteen models and it ranged between 4 and 16 layers [8–10, 15, 17, 19, 23, 28, 33] (Table 4).

**Table 4:** Characteristics of selected studies

Authors name (year of publication)	Title	Type of substrate	Type of Cells	Structure/ layers no.	Cell Markers expression	Model functionality
[10]	Development of a novel three-dimensional in vitro model of oral Candida infection	Rat tail collagen type I	Model (1) Primary human gingival keratinocytes and fibroblasts Model (2) Human OKF6/TERT-2 cells with human primary gingival fibroblasts	Model (1) keratinocytes showed a high degree of differentiation. Model (2) 1. (7 -12) cell layers of epithelial cell. 2.The basal layer invaded the submucosal compartment.		Following candida infection:- 1.Degradation of the cornified layer of epithelial cells, extensive cellular necrosis, and loss of cellular junctions in the stratum basale. 2.increased cytokine secretion IL-1a α.
[24]	Histomorphological and biochemical differentiation capacity in organotypic co-cultures of primary gingival cells	Rat tail collagen type I	Primary human gingival keratinocytes and fibroblasts	1.keratinocyte cells formed multilayered epithelium. 2.fibroblasts cells incorporated into collagen lattices.	1. CK14, CK4 and CK13. 2. some keratinocytes cells are sensitive to vimentin. 3. collagen type IV and laminin.	
[19]	The biological seal of the implant–soft tissue interface evaluated in a tissue engineered oral mucosal model	Acellular cadaveric dermis (Alloderm)	Human oral keratinocyte cell line (TR146) and human primary gingival fibroblasts	1. 50–100 mm thick, well-formed, stratified squamous epithelium of (4-6) epithelial layers 2.Well cells attached to the Ti surfaces and form a cell network on all the Ti surfaces		1-Normal permeability test for biological seal and cell attachment to Ti disc evaluation 2-Normal Alamar Blue assay test value of residual cells attached to the Ti discs.
[25]	Development of a Full-Thickness Human Gingiva Equivalent Constructed from Immortalized Keratinocytes and Fibroblasts	Rat tail collagen type I	Model (1) Primary human gingival keratinocytes and fibroblasts Model (2) Immortalized human gingiva Keratinocytes cell line, OKG4/bmi1 /TERT, The human gingiva fibroblast cell line was TERT immortalized (T0026) Model (3) Immortalized human gingiva Keratinocyte cell line, human papillomavirus type 16 (KC-HPV) and the human gingiva fibroblast cell line was TERT immortalized (T0026)	1- In both primary and keratinocytes TERT cells, a differentiated stratified epithelium on a fibroblast populated collagen hydrogel was observed and fibroblast-populated collagen was observed without deep rete ridges 2. Model constructed with KC-HPV did not form a well-differentiated epithelium with a disorganized multilayer was formed.	Model (1) 1.CK10, and K13 2. Involucrin 3. Ki67 4. Collagen type IV and laminin 5 Model (2) 1. very low expression of involucrin, K10, K13 protein and Ki67 2. collagen type IV and laminin 5	
[9]	An In-Vitro Analysis of Peri-Implant Mucosal Seal Following Photofunctionalization of Zirconia Abutment Materials	Acellular cadaveric dermis (Alloderm)	Primary human gingival keratinocytes and fibroblasts. + zirconia implant abutment	1.(4-6) layers of epithelial Cells 2. Model tissue was attached to the implant surface. 3- Long junctional epithelial attachment was observed in smooth titanium than in the rougher surface, whereas the rougher titanium surface had a long dimension of connective tissue attachment		Permeability test for a biological seal of tissues around Ti disc evaluation as normal.
[15]	Commensal and pathogenic biofilms differently modulate peri‐implant oral mucosa in an organotypic model	Bovine collagen type I	immortalized human oral keratinocyte cell line (OKF6/TERT‐2) and Primary human gingival fibroblast	1. (4) different layers of the differentiated epithelium, 2-Tight epithelial barrier 3 Model tissues were attached to the implant surface.		Following biofilm challenges: - increase in TNF-α and decrease of IL-6, CXCL8, CXCL1 and CCL2 inflammatory cytokine levels.
[34]	Establishment and Characterization of Immortalized Gingival Epithelial and Fibroblastic Cell Lines for the Development of Organotypic Cultures	Rat tail collagen type I	Immortalized human gingival (epithelial keratinocytes (HGEK-16) and fibroblasts (GFB-16)) were induced by E6 and E7 oncoproteins of human papillomavirus	1-Multi layered epithelium with no keratinizing of superficial layer 2- fibroblasts were evenly distributed in the Collagen gel matrix.	1- CK10, CK13, CK16, CK18, and CK19 2- Col I and Col II	
[35]	Phenotypic markers of oral keratinocytes seeded on two distinct 3D oral mucosa models	1-Rat tail collagen type I 2-Acellular cadaveric dermis (Alloderm) 3-Porcine acellular dermal matrices (Strattice)	Primary human gingival keratinocytes and fibroblasts	1-Rat tail collagen type I gingival fibroblasts presented homogeneous distribution and lower adhesion and differentiation of oral keratinocytes 2- AlloDerm and Strattice matrices fibroblasts adhered well to the dermal surface.	1-Glucose consumption, proliferation of gingival fibroblasts 2-synthesis of hVEGF 3-gene expression of COLIA1 and hVEGF 4- AlloDerm substrate provided higher values for cell proliferation, and both gene expression, synthesis of hEGF and hKGF by oral keratinocytes	
[21]	BMP4 micro-immunotherapy increases collagen deposition and reduces PGE2 release in human gingival fibroblasts and increases tissue viability of engineered 3D gingiva under inflammatory conditions	Rat tail collagen type I	Immortalized Human Gingival Keratinocytes (iHGK) and Immortalized Human Gingival Fibroblasts-hTERT	1-A good multilayer epithelial 2.fibroblasts embedded in the collagen matrix.	1.Involucrin, CK 19 and 17 2. Vimentin marker for fibroblast.	1- High MTT assay 2-Low measured of (LDH) activity.
[33]	Development and characterization of a 3D oral mucosa model as a tool for host-pathogen interactions	Rat tail collagen type I	NOK-si keratinocytes immortalized human oral keratinocytes cells and Primary human gingival Fibroblast cells	1- 6-8 layers of stratified epithelium tissue cells. 2-Fibroblasts and collagen fibres showed a structural arrangement forming an intricate network	1.CK 13 and 14. 2. Ki-67. 3.Collagen IV.	Destruction of epithelial layers after bacterial challenges.
[26]	Development and Characterization of In Vitro Human Oral Mucosal Equivalents Derived from Immortalized Oral Keratinocytes	Rat tail collagen type I	Model (1) FNB6-TERT immortalized human oral keratinocytes and Human Primary gingival fibroblasts cells Model (2) H357, an human oral squamous cell carcinoma (OSCC) cell line derived from the tongue and Human Primary gingival fibroblasts cells	Model (1) a multi-layered well-defined, stratified epithelium (120 µm) in thickness. The epithelium was stratified, nonkeratinized, Model (2) produced a multi-layered epithelium.	Model (1) a. ki-67. b. CK13. c. E-cadherin. d. CK14. e.Gene expression for CXCL8 and ICAM-1. Model (2) a. ki-67. b. E-cadherin. c. CK 13 and 14.	Increased secreassion of cytokines following bacterial challenge: - CXCL8 and IL-6
[8]	Limited in-depth invasion of Fusobacterium nucleatum into in vitro reconstructed human gingiva	Rat tail collagen type I	primary gingival keratinocytes and fibroblasts	(12–16) epithelial layers.	CK 13, CK19, and CK 10.	Destruction of epithelial layers after bacterial challenges.
[22]	Establishment of an oral infection model resembling the periodontal pocket in a perfusion bioreactor system	Porcine collagen, type I (3D collagen sponge)	Immortalized human gingival (epithelial keratinocytes (HGEK-16) and fibroblasts (GFB-16)) were induced by E6 and E7 oncoproteins of human papillomavirus	1- Well defined epithelial cell layers. 2- Fibroblast cells filled most gaps between collagen fibers and formed a dense structure.		Increased secretion of cytokines following bacterial challenge: - IL-1b, IL-2, IL-4, and TFN-a
[27]	Saliva-Derived Commensal and Pathogenic Biofilms in a Human Gingiva Model	Rat tail collagen type I	Immortalized human gingiva cell line (Keratinocytes OKG4/bmi1/ TERT and fibroblast TERT (T0026)	1.Multilayered differentiated epithelium. 2.fibroblast-populated collagen substrate.		1.Destruction of epithelial layers after bacterial challenges. 2. Increased secretion of cytokines following bacterial challenge: - CCL20, IL-6, CXCL8, and CCL2
[17]	Evaluation of a novel oral mucosa in vitro implantation model for analysis of molecular interactions with dental abutment surfaces	Rat tail collagen type I	Immortalized human gingiva keratinocyte (KC-TERT, OKG4/bmi1/TERT And fibroblast cell lines (Fib-TERT, T0026))	1- (7-9) layers of well differentiated stratified. 2.fibroblast-populated collagen. 3- epithelial down-growth. parallel to the surface of both abutments.	1. Ki67 2. A collagen IV/laminin V 3. CK 4 and 19	The interactions of gingival tissue to implant surface were similar to two types of titanium abutments, anodized and machined,
[20]	Multi-species oral biofilm promotes reconstructed human gingiva epithelial barrier function	Rat tail collagen type I	immortalized human gingiva keratinocyte (KC-TERT,OKG4/bmi1/TERT) and fibroblast (Fib-TERT, T0026) cell lines	Thick and multiple keratinocyte layers.	1. PCNA protein 2. Ki-67.	1.Increased thickness of epithelial layers after bacterial challenges. 2. Increased secretion of cytokines following bacterial challenge:- IL-6, CXCL8, CXCL1, CCL20.
[29]	Oral mucosa model based on a collagen–elastin matrix	Collagen/elastin matrix (Matriderm, bovine collagen type I with elastin)	Primary human gingival keratinocytes and fibroblasts.	1- Multilayered formation of gingival keratinocytes 2- Prominent basement membrane	collagen IV.	
[23]	Tissue engineering of human oral mucosa on different scaffolds: in vitro experiments as a basis for clinical applications	1-Dermal Regeneration Template (DRT) 2-Vicryl 3-TissuFoil E (TFE)	Human primary gingival keratinocytes and fibroblast.	1- DRT. Owing to the rough surface, fibroblasts were able to migrate into the scaffold with the seeding of keratinocytes and the epithelium formed 2.7 layers of keratinocytes. 2- On Vicryl, fibroblasts were able to grow as well as keratinocytes, but no stratification of cells was visible in the dermis (fibroblasts) and epidermis (keratinocytes) as occurred on TFE and DRT 3- On TFE demonstrated formation of epithelium with 9.3 layers of keratinocytes which formed a homogeneous stratified cell layer	1. Cells on DRT expressed more laminin 1 than cells on TFE 2. Collagen IV in TFE and DRT 3. On Vicryl, no collagen IV staining could be observed.	
[28]	In vitro reconstruction of human junctional and sulcular epithelium	Rat tail collagen type I	Model (1) Primary human gingival keratinocytes and fibroblasts Model (2) Primary human gingival keratinocytes and primary periodontal fibroblasts	(11–16) epithelial layers	1-Ki-67 2- ODAM 3-FDC-SP 4- CK 8, CK10, CK13, CK16, and CK19. 5-transglutaminase. 6- filaggrin. 7- collagen IV and Laminin-1.	
[18]	Differential influence of Streptococcus mitis on host response to metals in reconstructed human skin and oral mucosa	Rat tail collagen type I	Primary human gingival keratinocytes and fibroblasts	Thick and multiple keratinocyte layers	Ki67.	Increased expression of Toll-like receptors 4 following bacterial challenge.
[30]	3D engineered human gingiva fabricated with electrospun collagen scaffolds provides a platform for in vitro analysis of gingival seal to abutment materials	1-Electrospun bovine collagen type I 2-decellularized dermis 3-Bovine collagen type I type I 4-Released bovine type I collagen	Primary human gingival keratinocytes and fibroblasts	stratified epithelium with a layer of tightly packed basal keratinocytes was present along the junction between the epithelium and connective tissue.	1. CK4, CK5, CK10 2. collagen IV and laminin -332. 3.collagen type I	There were tissue attachments with the following implant surfaces: - 1. machined titanium. 2. SLA (sandblasted-acid etched) titanium. 3. ceramic. 4.PEEK (Polyethere-therketone).
[16]	Engineering a 3D In Vitro Model of Human Gingival Tissue Equivalent with Genipin/Cytochalasin D.	1- Rat tail collagen type I. 2- Rat tail collagen type I that crosslinked with genipin. 3- Rat tail collagen type I that crosslinked with cytochalasin D. 4- Rat tail collagen type I that crosslinked with genipin/ cytochalasin D,	Immortalized human gingiva keratinocyte hTERT (TIGKs, CRL-3397, ATCC) and Immortalized human gingiva fibriblast hTERT (hGFBs, CRL-4061, ATCC)	multilayered stratified epithelium with clear suprabasal and basal layers in the epithelium, similar to human native gingiva. The epithelium formed on the surface of collagen hydrogel populated with fibroblasts.	1. Ki67 2-CK14, and CK10, Involucrin. 3-vimentin, collagen 1a1, and CD9	1-The sizes of crosslinked models with genipin or genipin/ cytochalasin D were larger than non-crosslinked model and crosslinked model with cytochalasin D. 2-The size of crosslinked model with cytochalasin D was a bit larger than non-crosslinked model.

Dabija-Wolter et al. reported the number and thickness of epithelial layers. The thickness of epithelium at day 3 of development was 37.73 µm, and 49.79 µm, 130.93 µm, and 190.83 µm were at days 5, 7, and 9 respectively [28]. The study by Jennings et al. reported 120 µm thickness of well stratified epithelium. Chai et al. reported a pre-implant gingival model with a thickness of 50–100 µm [19] while Kriegebaum et al. demonstrated the formation of gingival model with an epithelium layer with 111.6 µm and 31 µm in thick when (TFE) and (DRT) were used respectively [23].

#### Connective tissue layer

With regards to the characteristic of connective tissue layer formation, eleven studies with nineteen models confirmed fibroblasts embedded in well-structured collagen fibrils [16, 17, 21, 23–25, 29, 30, 33–35]

Only one study reported the thickness of connective tissue layer, this study showed that by using TFE and DRT as substrates for gingival model construction the formation of connective tissue layers was 249.3 µm and 420.9 µm respectively [23].

### Differentiation of gingival model

Thirty models from sixteen studies reported several expression markers to evaluate biological structures included in the constructed models. The reported markers were K4, K5, K10, K13, K14, K16, K17, K18, K19, involucrin, laminin5, proliferation marker Ki67, CD90, and vimentin, Type I, II and IV collagen [8, 16–18, 20, 21, 23–26, 28–30, 33–35].

#### Keratinocytes proliferation marker

The expression of keratinocytes proliferation marker Ki67 was investigated in eleven models [8, 16–18, 20, 26, 27, 33]. In addition to Ki67, one study analyzed the expression of PCNA as a marker for cell proliferation, which also confirmed the proliferation potential of keratinocytes in the model [20]. In contrast, apoptotic p53 marker was not detected in models prepared by using collagen type I hydrogel [16].

#### Keratinocytes differentiation markers

##### Cytokeratins

Cytokeratins (CKs) are the main intermediate filaments of gingival epithelia. Within the gingiva, the expression patterns of various CKs have been used as molecular indicators for different oral gingival epithelium regions [36, 37].

CK4 is predominantly found in the suprabasal compartment of non-keratinized epithelia including the buccal mucosa of the sulcular gingival epithelium. Tomakidi et al. analyzed the expression of CK4 in models constructed using primary non-keratinized gingival cells where the positive expression of CK4 in suprabasal layer was observed [24]. Roffel et al. reported a peri-implant gingival model, and the expression of CK4 was observed in the free gingival epithelia and sulcular epithelium but not in the junctional epithelium [17]. Sakulpaptong et al. reported the expressions of CK4 were observed in peri-implant gingival models prepared from human primary gingival cells. In addition, the expression of CK4 in the human native gingival tissue was also reported in this study [30].

CK13, a marker for non-stratified epithelial, was investigated in eight studies [8, 15, 24–26, 28, 33, 34]. Buskermolen et al. showed the expression pattern of CK13 in the gingival model, constructed with both primary and immortalized gingival keratinocytes, was similar to native gingiva. The gingival model established with KC-HPV showed a very low expression of CK13 [25]. However, the study by Jennings et al. reported that the abnormal expression of CK13 was observed in the gingival model using OSCC cells [26].

CK14, a basal cell specific marker, was evaluated in four studies. Tomakidi et al. showed the expression of CK14 was only limited to the basal layer [24]. In contrast, de Carvalho Diasa et al. and Koskinen Holm, C., & Qu, C., reported the expression of CK14 in both basal and suprabasal layer [16, 33]. Jennings et al. observed the expression CK14 throughout the entire epithelium [26]. And Bao et al. reported lower levels of CK14 expressions in gingival models in comparison to the human gingiva tissue [34].

CK5 is generally found in the basal cell compartment in all stratified epithelia. Two studies investigated the expression of CK5 [24, 30] and reported its expression limited to the basal cell compartment as revealed by gene expression study as well as immunolocalisation study. In a study by Sakulpaptong et al. [30], CK5 was expressed in peri-implant gingival models as well as in human native gingival tissue.

CK10 is known to be largely expressed in cornifying stratified and proliferating epithelia. Six studies analyzed the expression of CK10 in gingival models [8, 15, 16, 24, 25, 34]. Buskermolen et al. and Koskinen Holm, C., & Qu, C., showed the expression pattern of CK10 in the gingival models were similar to native human gingiva. However, the expression of CK10 was at a very low level in the model made with immortalized cell KC-HPV [16, 25].

Other cytokeratins such as CK8, CK16, CK18, CK19 and CK17 were investigated only in three studies [21, 28, 34].

The expression levels of CK18 and CK19 were similar between 3D and native human gingival tissue [34]. The expression of CK17 and CK19 was confirmed to be expressed in keratinocytes at multilayer in the 3D model by Ferra-Cancellas [21]. Dabija-Wolter et al. reported the expressions of CK 16 were observed in the suprabasal layer of the gingival model, and in both parabasal and suprabasal layers in native human gingival tissue. In the same study, the expression of CK19 and CK8 was observed in all cell layers l. However, both markers were expressed in few patterns in the basal layer of human native gingival tissue [28].

##### Other keratinocytes differentiation markers

Two studies showed the expression pattern of involucrin in the 3D gingival model was similar to native human gingival tissue [16, 25]. Other markers such as ODAM, FDC-SP, transglutaminase, and filaggrin were reported as junctional epithelial-specific markers [28].

#### E-cadherin (epithelial cadherin)

E-cadherin is a major protein involved in cell-to-cell adhesion. The expression of E-cadherin was reported in three models [8, 15, 26], confirming the tight epithelial barrier.

#### Basement membrane markers

Collagen IV and laminin are important proteins within the basement membrane. Six studies investigated and confirmed the expression of these two proteins in the basement membrane in the models [17, 23–25, 28, 30].

#### ECM components collagen type I and collagen type II

In this review the expression pattern of collagen type I (Col I) and collagen type II (Col II) was reported in two studies, and the levels of expression were found not significantly differed from native human gingival tissue [30, 34]. However, one study reported expression of both collagen 1, and CD90 by using qRT-PCR technique [16].

#### Vimentin

Vimentin is a differentiation marker for fibroblast. Buskermolen et al. and Koskinen Holm, C., & Qu, C., showed the expression of this marker in gingival model to be similar to native gingival tissue. Similarly, Ferrà -Cañellas et al. reported the expression of vimentin in the gingival model, which confirms the development of fibroblast in the gingival model [16, 21, 25].

### Gingival model for periodontal research

**Fig. 2:**
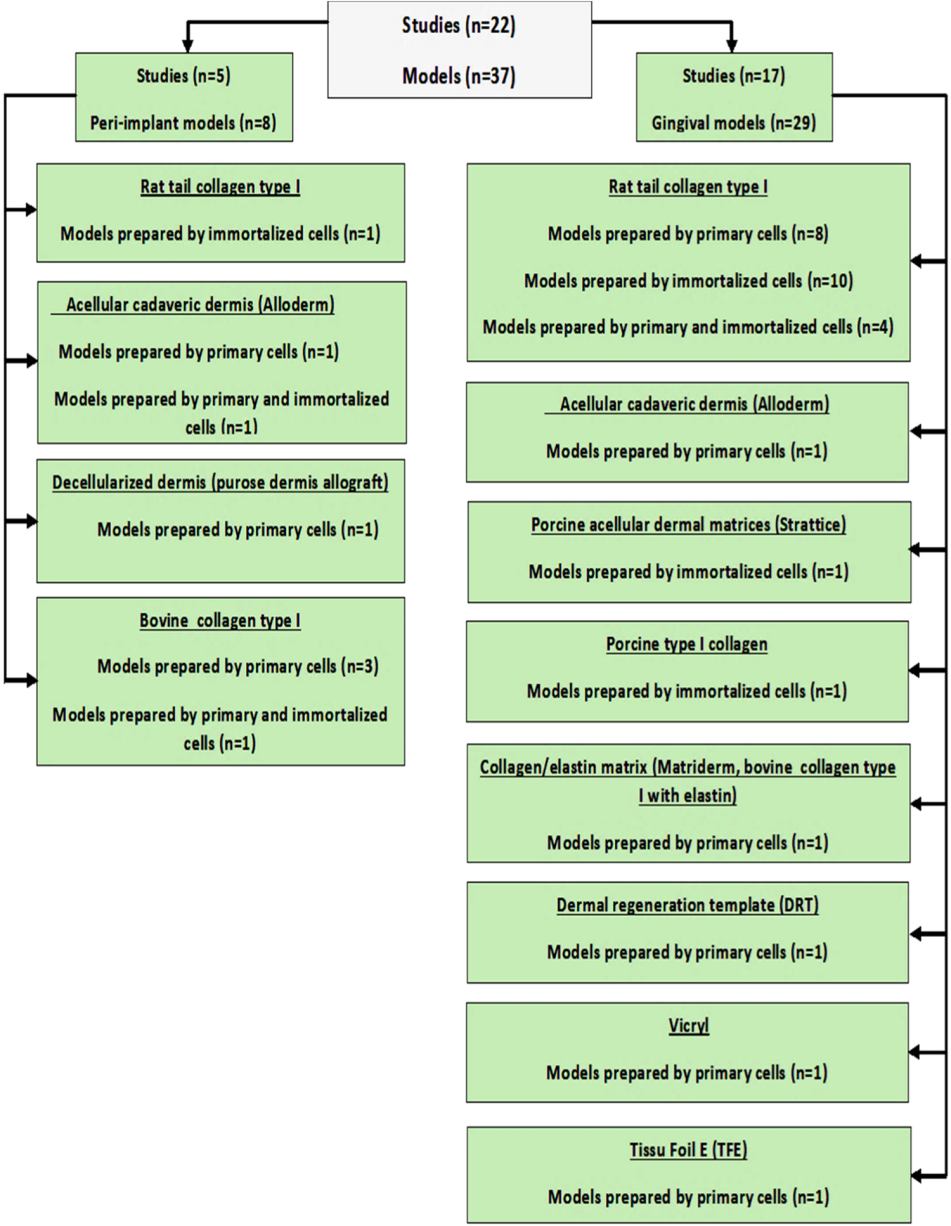
Flow chart of 3D gingival and peri-implant models

With regards to the application of these gingival models, studies demonstrated the utilization of these models in several periodontal research applications as well as eight peri-implant models used in five studies were found (Fig. 2).

#### Host and microbial interaction study

In total, nine studies demonstrated the applicability of gingival models in host-microbial interaction studies. Within these nine studies, seven studies reported the response of gingival models to different bacterial challenges [8, 18, 20–22, 26, 27]. Four studies [8, 20, 21, 27] demonstrated the alteration of the epithelial layer upon the host-microbial interaction.

Apart from host-microbial interaction, the gingival model was used to investigate candida infection and it showed alteration of the structure by prominent degradation of the cornified layer of epithelial cells [10].

#### Mucosal model for dental implant research

The peri-implant mucosal models were used either for comparing different types of titanium and dental material posts surfaces [17, 19, 30], or for photofunctionalized effect on the biological seal of different types of abutment materials [9].

#### Gingival model for periodontal wound healing and regeneration

Potential application of 3D gingival models to study wound healing processes of the gingiva either following cold injury [27], micro-immunotherapy medicine (low dose of bone morphogenic protein (LD BMP4)) [21], or for the exposed model to sensitizers (Lin [18]).

### Substrate biomaterials for construction of gingival model

In this review, 10 different substrate types were identified among all 37 models reported. The most used substrate was type I collagen sourced from rat tail, which was used in twenty-three models [8, 10, 17, 18, 20, 21, 24–28, 33–35]). Acellular human cadaveric dermis substrate (Alloderm) was used in three models [9, 19, 35], and decellularized dermis (purose dermis allograft) used in another model [30]. The other substrate including porcine collagen type I [22], porcine acellular dermal matrices (Strattice) [35], collagen/elastin matrix substrate (Matriderm) (bovine collagen type I with elastin) [29], dermal regeneration template (DRT) Single Layer substrate, Vicryl substrate, Tissu Foil E (TFE) [23], bovine type I collagen substrate were used to prepare four models [15, 30], (Table 4) and (Fig. 2).

## Discussion

This review comprehensively described thirty-seven different 3D gingival and peri-implant models from twenty-two research studies. Twelve of these models confirmed good cell proliferation (marker Ki67) in both basal and suprabasal layers and most of the models confirmed good differentiation of epithelial cells (reporting different CKs markers). This was the first attempt to collectively appraise the available evidence resulting in not a single better model to study and test 3D gingival or peri-implant tissues.

Several studies have constructed gingival models from different cell origins, including primary cells, immortalized cell lines or a mixture of both. The highest number of epithelial layers was reported from the model using the primary cells origin [8]. In this review, two models were prepared from immortalized cell lines, H357, and OSCC, and demonstrated to be deficient in a well-defined differentiated epithelium [25, 26]. In contrast, one study reported that established Immortalized cell lines from primary human gingival cell induced by E6 and E7 oncoproteins of human papillomavirus, and resulted in a successful formation of gingival model with multi-layered epithelia [34]. These observations confirmed that these two types of immortalized human gingival cells (H357 and OSCC) are not suitable sources for gingival model construction. Further this review highlighted that when using cell lines in 3D gingival model construction, greater clarity in the presentation of the results is needed, this is because cell lines generally inherit the characteristics of their parental primary tissue cells hence when used these cells may not accurately reproduce properties or responses of normal epithelial cells [10, 34].

A crucial element in the construction of a gingival model is the substrate that provides scaffolding for the cells. The ideal substrate should have a high level of biocompatibility, porosity, biostability, and mechanical properties. In this review ten different substrates demonstrated to be applicable as matrices to mimic native gingival ECM and most of them were of animal origin. Rat tail collagen type I isolated from rat tail tendon was the most used and confirmed to allow the formation of the highest number of epithelial layers [8, 10, 28, 33]. The stratification of epithelial layers indicates the development of a gingival model, at the same time, a high level of stratification of keratinocytes has been demonstrated when there is an underlying homogenous distribution of fibroblasts among substrates. Rat tail collagen is considered the major type of collagen that is used as a substrate to mimic human ECM. Unfortunately, shrinkage is considered a disadvantage of models prepared by using collagen type I. This shrinkage can lead to a drastic decrease in the size of cell population in the hydrogel. However, it was reported that using genipin and genipin/cytochalasin D to crosslink collagen type I hydrogel allowed the construction of a model with more resistance to shrinkage facilitating in turn high cells survival and function [16]. Lastly, additional drawbacks for this collagen include its cost and its differences with human ECM´s collagen (where type I and III collagens are present as major constituents) plus isolated rat tail collagen is invariably fragmented [38]. All these drawbacks prevent considering rat tail collagen hydrogel to be ideal for gingival model construction.

Two more animal type of substrates were identified. A bovine collagen type I [15, 30] which demonstrated stratification and differentiation of epithelial layers with underlying connective tissue containing fibroblasts and a porcine substrate as a source of collagen type I to mimic human ECM as 3D collagen sponge scaffolds in a perfusion bioreactor system for easy manipulation [22]. However, these two substrates were not counted as a promising type for model construction due to lacking resemblance to native gingival human connective tissue.

In addition to collagen, dermal substrates were also widely used for tissue engineering and cell culture experiments. In this review, four dermal substrates were used for reconstructing gingival models including acellular cadaveric dermis and decellularized dermis (porous dermis allograft) as well as human [9, 19, 30, 35], porcine (strattice matrix) [35], and bovine (Matriderm) [29] sources. All of these dermal substrates showed good proliferation, differentiation and stratification of keratinocytes with a high distribution of fibroblasts. However, these types of substrates suffer from limited availability.

Lastly DRT was used as substrate for gingival model construction, a porous matrix of fibers of crosslinked bovine tendon collagen. High thickness tissue layers of gingival model with higher cells proliferation when compared to equine (TissuFoil E) and synthetic materials (Vicryl) substrate [23]. Electrospun type I crosslinked bovine collagen was used in one study to recreate a peri-implant gingival model [30] resulting in less tissue contraction and promising results. Size changes and contraction that occurred after model construction are attributed to the slow remodelling activity of the used substrates compared with native gingival tissue. This drawback is added to others mentioned above to take into account for proper selection of substrate to construct a developed gingival model.

It is worth mentioning that all the evidence on the use of different substrates collectively confirmed a high level of heterogeneity and the lack of a clear superior substrate to use for constructing the best 3D gingival model.

## Limitations and future research

This review highlighted high heterogeneity, and lack of standardized fabrication and characterization protocols for the creation of a valid 3D gingival or peri-implant model. As such, a new framework for future characterization and construction of a 3D gingival model should be proposed that accounts for the uncertainty identified within this study.

The first step should include histological confirmation that the new model results in well-defined stratified epithelium layers with equal or more than four cell layers, and fibroblasts embedded and distributed homogenously in a well-structured substrate. Secondly well differentiated tissue layers should be confirmed via specific markers expression for each cell or layer regions, as following:

- Ki67 for cell proliferation near basal epithelial layer
- CK14 and CK5 for early differentiation in the basal layer and CK4 or CK13 in the suprabasal layer.
- CK16, CK18, CK19 and CK17 in different epithelial layers as late differentiation markers
- Involucrin as terminal differentiation marker for keratinocytes within the upper two third of the epithelium
- CK10 marker to confirm the presence of cornifying stratified epithelia as well as in proliferating epithelia
- Collagen IV and Laminin expression for the basement membrane
- CD90 and Collagen (I and II) in ECM
- Vimentin expression to confirm development of fibroblasts.

**Fig. 3:**
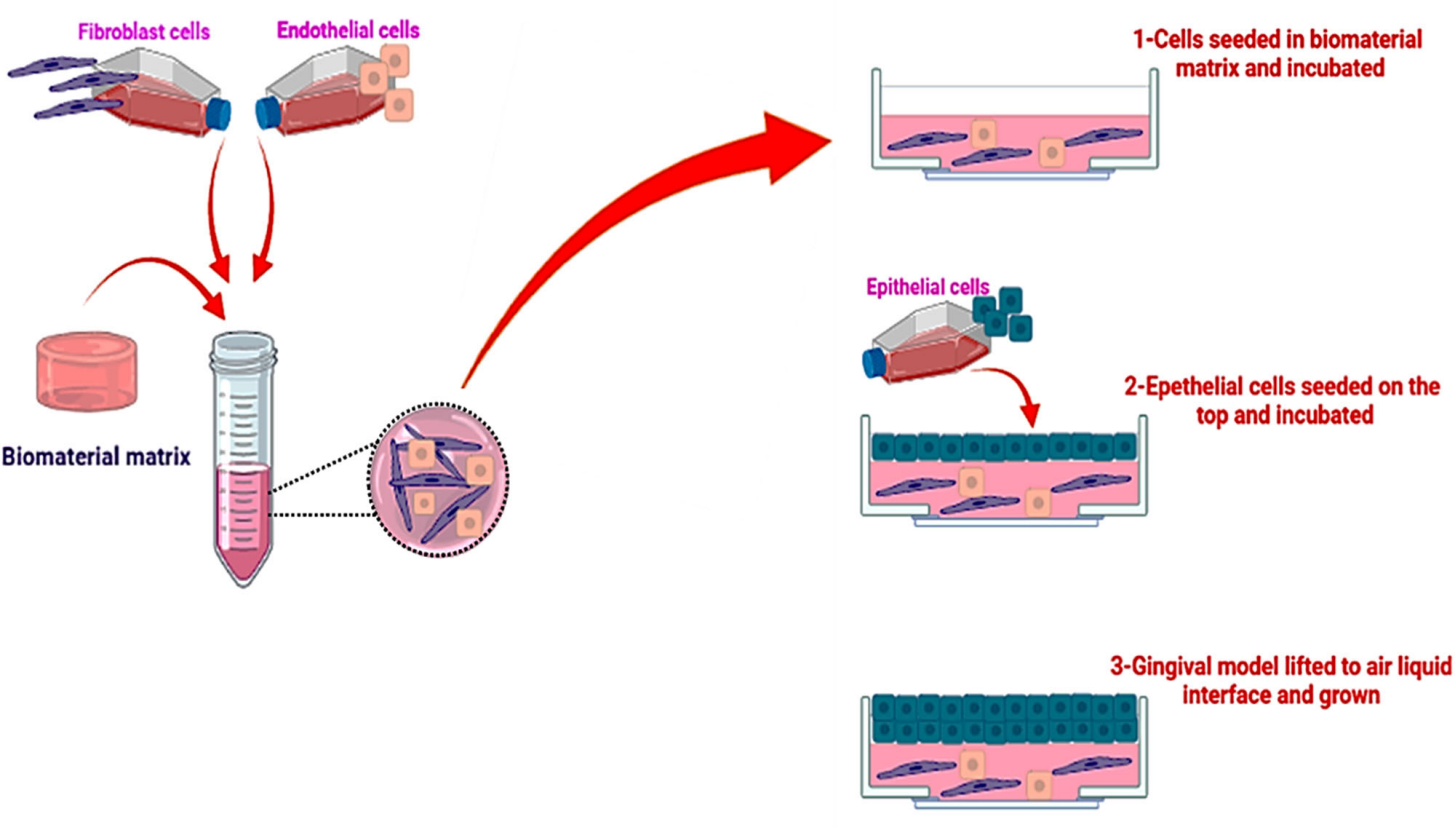
Schematic representation of the experimental protocol to generate 3D gingival model

Thirdly an ideal 3D gingival model to use for different dental applications will need a well-developed vascular structure including capillary vessels, epithelial and stromal cells as well as immune, neural and bone cells (Fig. 3).

## Conclusions

There is insufficient evidence to suggest whether the available 3D gingival models can entirely recapitulate the human gingival tissue and be valuable when performing experimental periodontal research. This review highlighted the lack of specific cell origin or substrate for constructing gingival models to reproduce physiologic properties of native human gingival tissue structures. Future research should aim at resolving the current challenges of construction a developed vascularized 3D gingival model mimic native human gingival tissue by engineering a new substrate with a high remodeling activity and suitable microenvironment for seeding human gingival cells.

### Supplementary Information

Zainab 3D model coverletter (resubmit, JMS)

## Data Availability

All data used to support the findings of this study are included within the article. Specifically, the registered systematic review protocol can be found at the Open Science Framework (OSF) database, hosted by the Center for Open Science(COS) (https://archive.org/details/osf-registrations-6mzw2-v1.
