# Supplementary material for: Three dimensional (3D) gingival models in periodontal research: a systematic review
Source: J Mater Sci Mater Med. 2023 Nov 8;34(11):58. doi: 10.1007/s10856-023-06761-z (PMC10632299; doi:10.1007/s10856-023-06761-z)
Supplement: Supplementary file 1 — Zainab 3D model coverletter (resubmit, JMS) [file 10856_2023_6761_MOESM1_ESM.docx]

**Supplemental Data 2. Categories used for quality assessment and risk of bias (modified from the ARRIVE and CONSORT guidelines) (Ramamoorthi, Bakkar, Jordan, & Tran, 2015)**

| Items | Description | Grade |
| --- | --- | --- |
| 1 | Title | (0) Inaccurate/no concise  (1) Concise/adequate |
| 2 | Abstract: either a structured summary of background, research objectives, key experiment methods, principal findings, and conclusion of the study or self-contained (should contain enough information to enable a good understanding of the rationale for the approach) | (1) Clearly inadequate  (2) Possibly accurate  (3) Clearly accurate |
| 3 | Introduction: background, experimental approach, and explanation of rationale/hypothesis | (1) Insufficient  (2) Possibly sufficient/some information  (3) Clearly meets/sufficient |
| 4 | Introduction: preprimary and secondary objectives for the experiments (specific primary/secondary objectives) | (1) Not clearly stated  (2) Clearly stated |
| 5 | Methods: study design explained number of experimental and control groups, steps to reduce bias (demonstrating the consistency of the experiment (done more than once), sufficient detail for replication, blinding in evaluation, etc.) | (1) Clearly insufficient  (2) Possibly sufficient  (3) Clearly sufficient |
| 6 | Methods: precise details of experimental procedure (i.e., how, when, where, and why) | (1) Clearly insufficient  (2) Possibly sufficient  (3) Clearly sufficient |
| 7 | Methods: How sample size was determined (details of control and experimental group) and sample size calculation. | (1) No  (2) Unclear/not complete  (3) Adequate/clear |
| 8 | Methods: Details of statistical methods and analysis (statistical methods used to compare groups) | (1) No  (2) Unclear/not complete  (3) Adequate/clear |
| 9 | Results: explanation for any excluded data, results of each analysis with a measure of precision as standard deviation or standard error or confidence interval | (1) No  (2) Unclear/not complete  (3) Adequate/clear |
| 10 | Discussion: interpretation/scientific implication, limitations, and generalizability/translation | (0) Clearly inadequate  (1) Possibly accurate  (2) Clearly accurate |
| 11 | Statement of potential conflicts and funding disclosure | (0) No  (1) Yes |
| 12 | Publication in a peer-review journal | (0) No  (1) Yes |
